# Activation of aryl hydrocarbon receptor by 6‐formylindolo[3,2‐b]carbazole alleviated acute kidney injury by repressing inflammation and apoptosis

**DOI:** 10.1111/jcmm.16168

**Published:** 2020-12-06

**Authors:** Sibei Tao, Fan Guo, Qian Ren, Jing Liu, Tiantian Wei, Lingzhi Li, Liang Ma, Ping Fu

**Affiliations:** ^1^ Division of Nephrology National Clinical Research Center for Geriatrics Kidney Research Institute West China Hospital of Sichuan University Chengdu China

**Keywords:** acute kidney injury, apoptosis, aryl hydrocarbon receptor, inflammation, renal tubular epithelial cell

## Abstract

Acute kidney injury (AKI) is a multifactorial disease of various aetiologies. Aryl hydrocarbon receptor (AhR) is a ligand‐activated transcription factor that responds to ligands to induce or repress gene expressions, thereby regulating a diverse spectrum of biological or pathophysiologic effects. However, the effect of AhR on AKI remains unknown. A single intraperitoneal injection of 50% glycerol was performed to induce rhabdomyolysis in C57BL/6J mice. The bilateral renal pedicles were occluded for 30 minutes and then removed to stimulate renal I/R injury. 6‐formylindolo[3,2‐b]carbazole (FICZ), a photo‐oxidation product of tryptophan with a high affinity for AhR, was used. The in vitro study was performed on HK‐2 cells. Ferrous myoglobin and FICZ was dissolved in the medium in different cell groups. Treatment with AhR agonist FICZ significantly alleviated the elevation of serum creatinine and urea in AKI. AKI modelling‐induced renal damage was attenuated by FICZ. AhR mainly expressed in proximal tubular cells and could be activated by FICZ administration. Meanwhile, AKI triggered the production of pro‐inflammatory cytokines in injured kidneys, while FICZ inhibited their expressions. Furthermore, FICZ effectively reversed cell apoptosis in AKI models. Mechanistically, AKI stimulated the activation of NF‐κB and JNK pathways in the kidneys, while FICZ significantly suppressed these corresponding protein expressions. For the in vitro study, FICZ also inhibited inflammation and apoptosis in myoglobin or H/R‐stimulated HK‐2 cells. In summary, agonism of AhR by FICZ alleviated rhabdomyolysis and I/R‐induced AKI. FICZ inhibited inflammation and apoptosis via suppressing NF‐κB and JNK pathways in proximal tubular cells.

## 1 INTRODUCTION

Acute kidney injury (AKI) refers to the rapid decline of renal function in a few hours to several days due to renal perfusion insufficiency, nephrotoxic drugs or poisons, urinary tract obstruction and other causes.^1^ The clinical causes of AKI mainly include ischaemia‐reperfusion injury, nephrotoxic substances (such as drugs, contract agent and muscle cell contents released by rhabdomyolysis) and sepsis. About 13.3 million people suffer from AKI every year in the world.^2^ AKI, with its high mortality and costs, brings heavy burden.^3^ Main pathophysiological mechanisms include the injury of renal vascular system,^4^, ^5^ endoplasmic reticulum stress,^6^, ^7^ mitochondrial damage,^8^ apoptosis,^9^ oxidative stress and inflammation.^10^ Rhabdomyolysis (RM) is a syndrome characterized by muscle necrosis and release of muscle cell contents into the circulation, during which AKI is the most common complication^11^; Ischaemia‐reperfusion (I/R) is one of the major pathophysiology of AKI, accounting for 60% of all AKI causes.^12^ At present, there is no effective treatment for AKI caused by both above‐mentioned reasons except for removing aetiology, supportive treatment and renal replacement therapy.

Aryl hydrocarbon receptor (AhR) is transcripted and translated from AHR gene. In humans, AhR is expressed in various organs, including lung, thymus, kidney, liver, and intestine, etc.^13^ Unliganded AhR resides in the cytosol in a protein complex also comprising a dimer of heat shock protein 90 (HSP90) and the co‐chaperones AIP and p23.^14^ Upon binding to a ligand, AhR is activated and enters the nucleus, where it binds to AhR nuclear translocator (ARNT), and promotes the transcription of many different downstream genes.^15^ Ligands for AhR are extremely diverse, and could be classified as exogenous or endogenous. Ligands with different sources and structures bind to AhR with different affinities and could trigger different physiological or pathological effects.^16^ Previous studies showed that AhR with its downstream pathways had the functions of anti‐inflammation,^17^ anti‐apoptosis,^18^ regulating cell cycles^19^ and regulating immunity,^20^ which are important components of the pathogenesis of AKI. Endogenous ligands for AhR are mainly from tryptophan metabolism. 6‐formylindolo[3,2‐b]carbazole (FICZ), a photo‐oxidation product of tryptophan, has a high affinity with AhR.^21^ Previously, FICZ exhibited protective effects in various disease models.^21^, ^22^, ^23^, ^24^ Although investigators found that activated AhR by uraemic toxins had adverse effects in patients with chronic kidney diseases (CKD),^25^ no study measured its changes of expression in AKI. It is also unclear whether FICZ has renal protective effect in rhabdomyolysis and I/R‐induced AKI, since AhR could activate totally different downstream effects.

## 2 MATERIALS AND METHODS

### 2.1 Chemicals, antibodies and primer sequences

Glycerol was purchased from Sigma. FICZ (SML1489, Sigma) was dissolved in 2.5% PEG400 and further diluted in normal saline prior to use. Antibodies and primer sequences were exhibited in Tables S1 and S2.

### 2.2 Animal experiments

All experimental protocols and animal procedures were conducted in accordance to the Ethical Principles in Animal Experimentation adopted by the Sichuan University of Experimentation and approved by the Animal Care and Use Ethics Committee of Sichuan University. Male C57BL/6 mice (10‐12 weeks, weight between 25 and 27 g) were purchased from Animal Laboratory Center of Sichuan University (Chengdu, China). The mice were kept under constant temperature (23 ± 2°C) and a light/dark conditions (12/12 hours), with free access to water and food. The mice were adapted to this environment for 1 week before further research.

In the rhabdomyolysis model, the mice were randomly divided into four groups: control (n = 6), FICZ (n = 3), glycerol (n = 6) and glycerol + FICZ (n = 6). The mice in the glycerol group were injected with 50% glycerol dissolved in 0.9% normal saline (9 μL/g body weight) into back limbs bilaterally to provoke rhabdomyolysis‐induced AKI. Meanwhile the mice in the control group received the same volume of saline by intramuscular injection. As for the glycerol + FICZ group, FICZ was dissolved in 2.5% PEG400 and diluted in 0.9% normal saline to be intraperitoneally given at a dose of 100 μg/kg/d for four consecutive days before glycerol injection. The mice in the FICZ group were administered intraperitoneally the same doses of FICZ for four consecutive days, without glycerol injection.

In the renal ischaemia‐reperfusion model, the mice were randomly divided into four groups: control (n = 6), FICZ (n = 3), I/R (n = 6) and I/R + FICZ (n = 10). The injury in the I/R group was elicited by clamping renal pedicles with non‐traumatic microvascular clamps for 30 minutes, following with 24 hours reperfusion after clamp removal. Occlusion and reperfusion were confirmed by colour changes of the kidneys. The mice in the control group underwent the same surgical procedures except for clamping pedicles. In terms of I/R + FICZ group, FICZ was administered as mentioned above for four consecutive days prior to I/R modelling. Similarly, the mice in the FICZ group were given the same doses of FICZ for four consecutive days, and experienced all surgical procedures as the control group.

Two batches of mice were respectively euthanized through pentobarbital sodium injection (50 mg/kg, ip) and sacrificed at 24 hours after modelling. The serum and kidney samples were collected and stored −80°C for further analysis.

### 2.3 Serum biochemistry assays

Blood samples were immediately centrifuged 1509.3 *g* for 20 minutes to isolate serum for testing. The serum creatinine (sCr), urea and creatine kinase (CK) levels were detected by automatic biochemical analyser (Mindray BS‐240). The sCr levels of the modelling groups rising up to two times of their control values were indicative of successful AKI modelling.

### 2.4 Histological evaluation

Kidney tissues were fixed in 10% neutral buffered formalin, embedded in paraffin and sectioned at 4 μm thickness. The sections were stained with periodic acid‐Schiff (PAS) after deparaffinization and rehydration. PAS‐stained sections were investigated under a light microscopy at magnifications of ×200 or ×400. Ten fields were randomly selected at a magnification of ×200 from each section and two sections were randomly selected from each sample of at least three in every group for semiquantitative analysis. Histopathological changes were assessed by the percentage of injured/damaged renal tubules, as indicated by tubular dilation, lysis, disruption and cast formation. Tissue injury was scored on a scale of 0‐4, with 0, 1, 2, 3 and 4 corresponding to 0%, <25%, 26%‐50%, 51%‐75% and >76% of injured/damaged renal tubules, respectively.^26^

### 2.5 Immunofluorescence staining

Mouse frozen kidney tissues, embedded in OCT, were cut into 4‐μm‐thick sections on a cryostat and stored at −80°C until staining. Phosphate‐buffered saline (PBS) containing 5% horse serum was applied for 1 hour at room temperature to block non‐specific binding sites. Then the sections were incubated with primary antibodies in a humidified chamber at 4°C overnight. After washing with PBS, the corresponding secondary antibody was used for 1 hour, avoiding light. The samples were then washed by PBS, stained with DAPI (D8200, Solarbio) and finally mounted with cover lips. Primary antibodies were replaced by PBS in negative controls. Secondary antibodies (1:500 dilution; Jackson ImmunoResearch) matched with primary antibodies were applied to export fluorescent signals. Images were captured and analysed by ZEN 2012 (blue edition) microscopy software.

### 2.6 Immunohistochemistry

Kidney tissues were formalin‐fixed, dehydrated, embedded in paraffin, sectioned at 5 μm thickness, and afterwards mounted on glass slides. The slides were then blocked by 2.5% normal goat serum and incubated with primary antibodies at 4°C. The slides were washed thrice in PBS, and we used VECTASTAIN ABC Kit (Vector) for staining. Images were captured using ZEN 2012 (blue edition) microscopy software.

### 2.7 TUNEL staining

Kidney tissues were formalin‐fixed, embedded in paraffin and sectioned at 4 μm thickness. The terminal deoxynucleotidyl transferase‐mediated dUTP nick end labelling (TUNEL) staining was performed using the DeadEnd™ Fluorometric TUNEL System (G3250, Promega). For nuclear staining, sections were incubated with DAPI (D8200, Solarbio) at a dilution of 1:500. Images were presented by fluorescence microscopy at magnifications of ×400. We counted positive cells and examined at least 10 fields per section in each sample.

### 2.8 Western blot analysis

The kidney tissues or HK‐2 cells were homogenized with radio immune precipitation (RIPA) lysis buffer (P0013B, Beyotime Biotechnology) containing 1‰ proteinase inhibitors (Keygen Biotech). After centrifugation (18 894.2 *g*, at 4°C) for 15 minutes, the supernatant was collected, and protein concentrations were determined by a bicinchoninic acid Protein Assay Kit (Beyotime Institute of Biotechnology) according to the manufacturer's instructions. Protein lysate with equal amounts were separated by 10%‐12% SDS‐PAGE followed by transfer onto polyvinylidene difluoride Membrane (Bio‐Rad). The membranes were blocked with 5% non‐fat dry milk (w/v) in Tris‐buffered saline with 0.1% Tween‐20 (TBS‐T) for 1 hour at room temperature and then incubated with corresponding primary antibodies overnight at 4°C. After being rinsed thrice with TBS‐T at 5‐minute intervals, the membranes were incubated with HRP‐conjugated secondary antibodies at room temperature for 1 hour. The protein blots on the membranes were visualized by the Immobilon Western Chemiluminescent HRP Substrate (Millipore Corporation) with Bio‐Rad Chemi Doc MP. Densitometry analysis was performed using ImageJ 6.0 software (National Institutes of Health) and β‐actin was used as a loading control.

### 2.9 Quantitative real‐time PCR analysis

Total RNA from frozen kidney samples was isolated with Animal total RNA isolation kit (Foregene). The concentration of isolated mRNA was determined by using a ScanDrop 100 (AnalytikJena) determiner and cDNAs were synthesized by using PrimeScript™ RT reagent kit (Takara Bio). Quantitative real‐time PCR was performed using iTaq™ Universal SYBR Green Supermix (Bio‐Rad) in a PCR system (CFX Connect; Bio‐Rad) after reverse transcription. Primer sequences are listed in Table S1. The relative gene quantities were calculated by the 2^−∆∆Ct^ method in comparison with the expression levels of β‐actin.

### 2.10 Cell culture and treatment

Human renal proximal tubular cell line (HK‐2 cell) was a kind gift from Prof. Xueqing Yu (Guangdong Provincial People's Hospital) and cultured in (DMEM)/F12 medium (SH30023. 01B, Hyclone) supplemented with 10% foetal bovine serum (SH30084. 03, Hyclone), at 37°C under humidified atmosphere of 5% CO_2_ and 95% air.

In the myoglobin model, the cells in exponential growth state were divided into four groups: the FICZ group, incubated with 100 nmol/L FICZ for 24 hours; the myoglobin group, incubated with 200 μmol/L ferrous myoglobin for 24 hours; the Mb + FICZ group, incubated with FICZ at 100 nmol/L with the administration of myoglobin treatment; and control group, cells were incubated with complete medium alone in the control group. Ferrous myoglobin was prepared as previously described.^26^, ^27^

In the hypoxia/reoxygenation (H/R) model, we divided the HK‐2 cells into three groups. The cells in H/R group were cultured under hypoxic conditions (1% O_2_, 94% N_2_ and 5% CO_2_) in medium without serum for 12 hours to induce hypoxia. Then the cells were transferred back to regular culture medium with oxygen for 6 hours for reoxygenation. Control cells were incubated in complete culture medium in a regular incubator. FICZ administration (100 nmol/L) in the H/R + FICZ group was given just before H/R operation.

### 2.11 Annexin V‐FITC/Propidium iodide assay

We applied the Annexin V‐FITC apoptosis analysis kit (AO2001‐02P‐H, SUNGENE). HK‐2 cells were seeded in six‐well plates. After being exposed to different media for 24 hours, the adhered cells were detached by trypsin. Then, the cells were resuspended in a binding buffer and stained with 5 μL of Annexin V‐FITC and 5 μL of propidium iodide (room temperature, 15 minutes, avoiding dark). Apoptotic cells were identified by a flow cytometer.

### 2.12 Statistical analysis

Data were given as mean ± SD. Statistical values for multiple group comparisons were analysed by one‐way ANOVA, and pairwise comparison was conducted by SNK test. All statistical analyses were performed in SPSS 21.0. *P* < 0.05 was considered as statistically significant.

## 3 RESULTS

### 3.1 AhR agonist FICZ attenuated AKI

**Figure 1:**
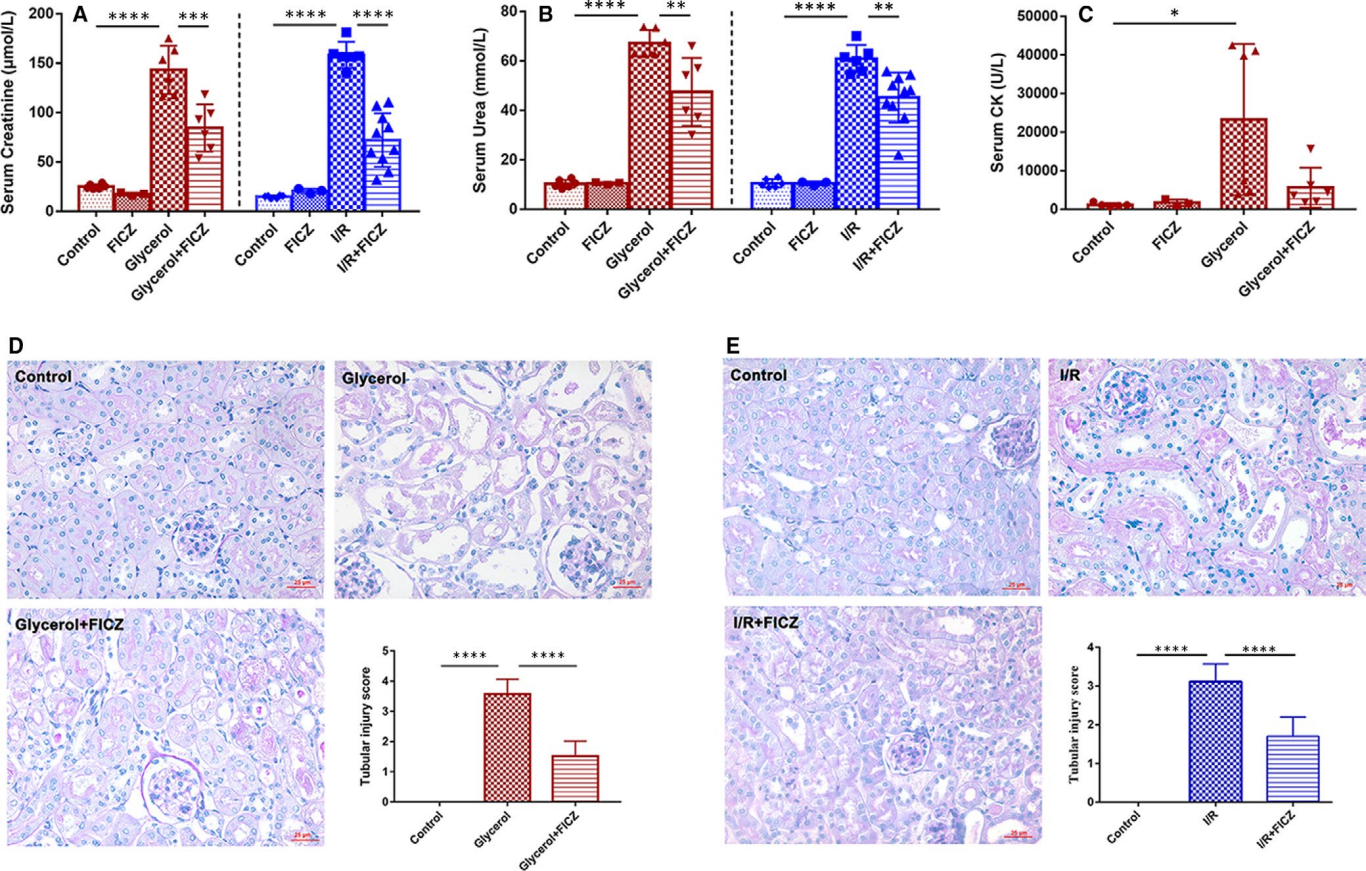
6‐formylindolo[3,2‐b]carbazole (FICZ) alleviated AKI. A, serum creatinine, B, urea and C, CK changes. D, Photomicrographs illustrate PAS (×400) staining with tubular injury scores of kidney tissues in rhabdomyolysis and E, I/R‐induced AKI. ****P < 0.0001, ***P < 0.001, **P < 0.01, *P < 0.05

To verify whether FICZ provides renoprotective effect, we established rhabdomyolysis and ischaemia/reperfusion (I/R)‐induced AKI models. Then we assessed renal function and pathological changes in kidney tissues. No significant perturbations in serum levels of creatinine, urea, CK, metabolic activities of liver enzymes in FICZ group were observed (Figure 1A‐C; Table S3). As shown in Figure 1A‐B, FICZ administration significantly reduced the remarkable rise of serum creatinine and urea levels at 24 hours after glycerol injection or I/R surgery, without influencing the serum CK level in rhabdomyolysis mice (Figure 1C). Meanwhile, PAS stainings displayed FICZ effectively alleviated pathological damage characterized by tubular dilation, swelling, cast formation and loss of brush border compared to that of AKI models in both models (Figure 1D‐E).

**Figure 2:**
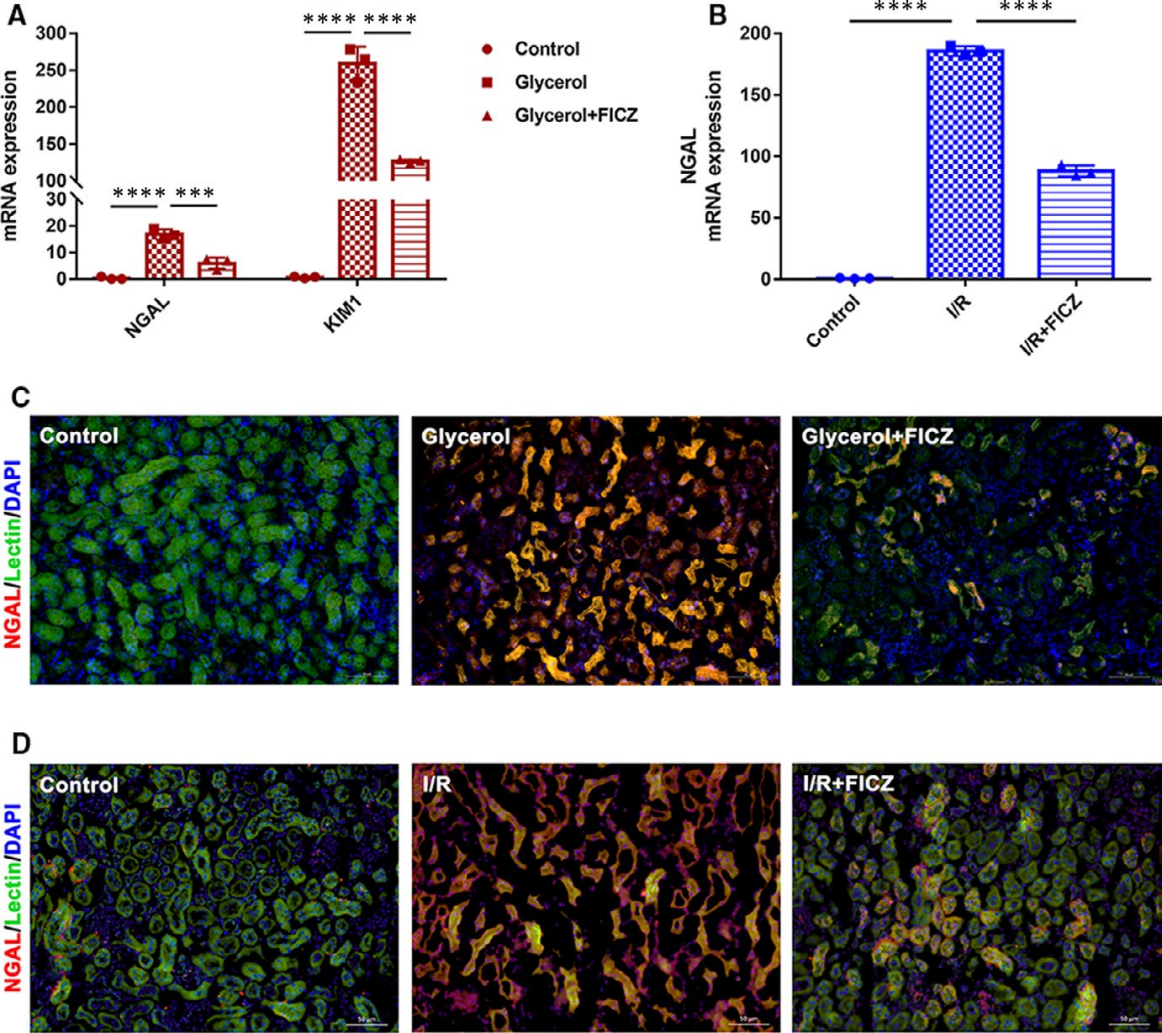
6‐formylindolo[3,2‐b]carbazole (FICZ) inhibited NGAL and KIM1 in kidneys. A, Quantitative real‐time PCR analysis of NGAL and KIM1 in renal tissues in rhabdomyolysis‐induced AKI. B, Quantitative real‐time PCR analysis of NGAL in renal tissues in I/R‐induced AKI. C, Immunofluorescence staining (×200) of NGAL and Lectin in the kidney tissue sections in rhabdomyolysis and D, I/R‐induced AKI. ****P < 0.0001, ***P < 0.001

Immunofluorescence staining and real‐time PCR results exhibited that use of FICZ significantly reduced the expression and transcription of neutrophil gelatinase‐associated lipocalin (NGAL), a renal injury biomarker, in the injured kidneys (Figure 2A‐D). Similarly, FICZ also inhibited glycerol‐induced mRNA level of kidney injury molecule 1 (KIM1) after the injection of glycerol (Figure 2A).

**Figure 3:**
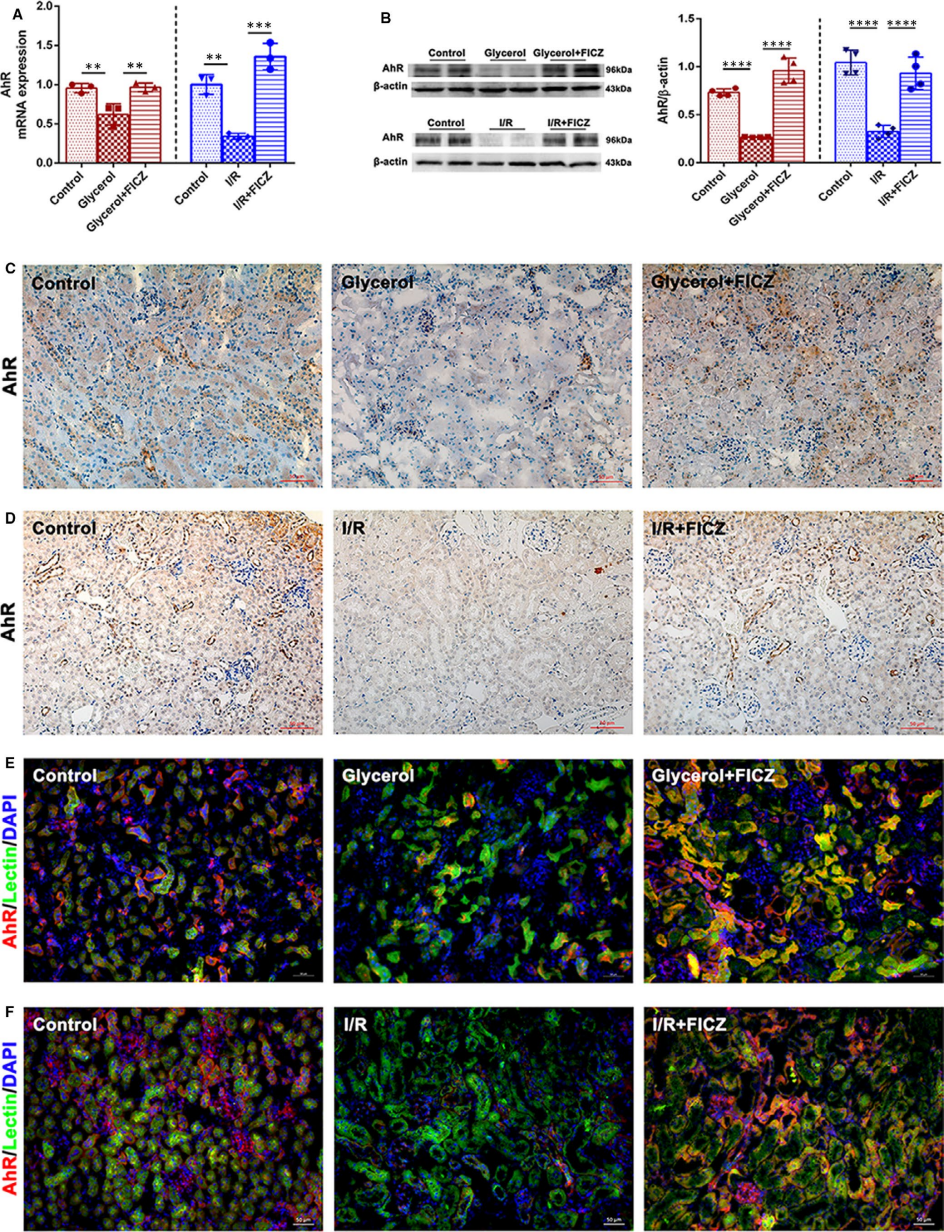
The mRNA and protein levels of AhR in kidney tissues. A, Quantitative real‐time PCR analysis of AhR in kidneys in rhabdomyolysis and I/R‐induced AKI. B, Immunoblot analysis of AhR and quantifications by densitometry normalized with β‐actin in kidneys. C, Immunochemistry staining (×200) of AhR in kidney tissue sections in rhabdomyolysis and D, I/R‐induced AKI. E, Immunofluorescence (×200) of AhR and Lectin in kidney tissue sections in rhabdomyolysis and F, I/R‐induced AKI. ****P < 0.0001, ***P < 0.001, **P < 0.01, *P < 0.05

We further examined the transcription and expression of AhR in kidney tissues. In Figure 3, the results of rt‐PCR, western blotting, immunohistochemistry and immunofluorescence staining inhibited that the mRNA and protein levels of AhR was significantly reduced in both AKI modelling groups. Intraperitoneal injection of FICZ significantly stimulated its transcription and expression in glycerol + FICZ and I/R + FICZ groups. As renal tubular epithelial cells play vital role in AKI,^28^ we tested whether AhR could be expressed in tubular epithelial cells. Immunochemistry results showed that AhR was mainly expressed in renal tubules (Figure 3C‐D). Besides, kidney tissue sections were stained with lectin, a marker for proximal tubular epithelial cells, AhR and DAPI for nuclei. As displayed in Figure 3E‐F, AhR merged with lectin, thus indicated that AhR mainly expressed in proximal tubular epithelial cells. Taken together, FICZ protected against rhabdomyolysis and I/R‐induced AKI via the activation of AhR in the injured kidneys.

### 3.2 FICZ reduced pro‐inflammatory cytokine production in the kidneys of AKI mice

**Figure 4:**
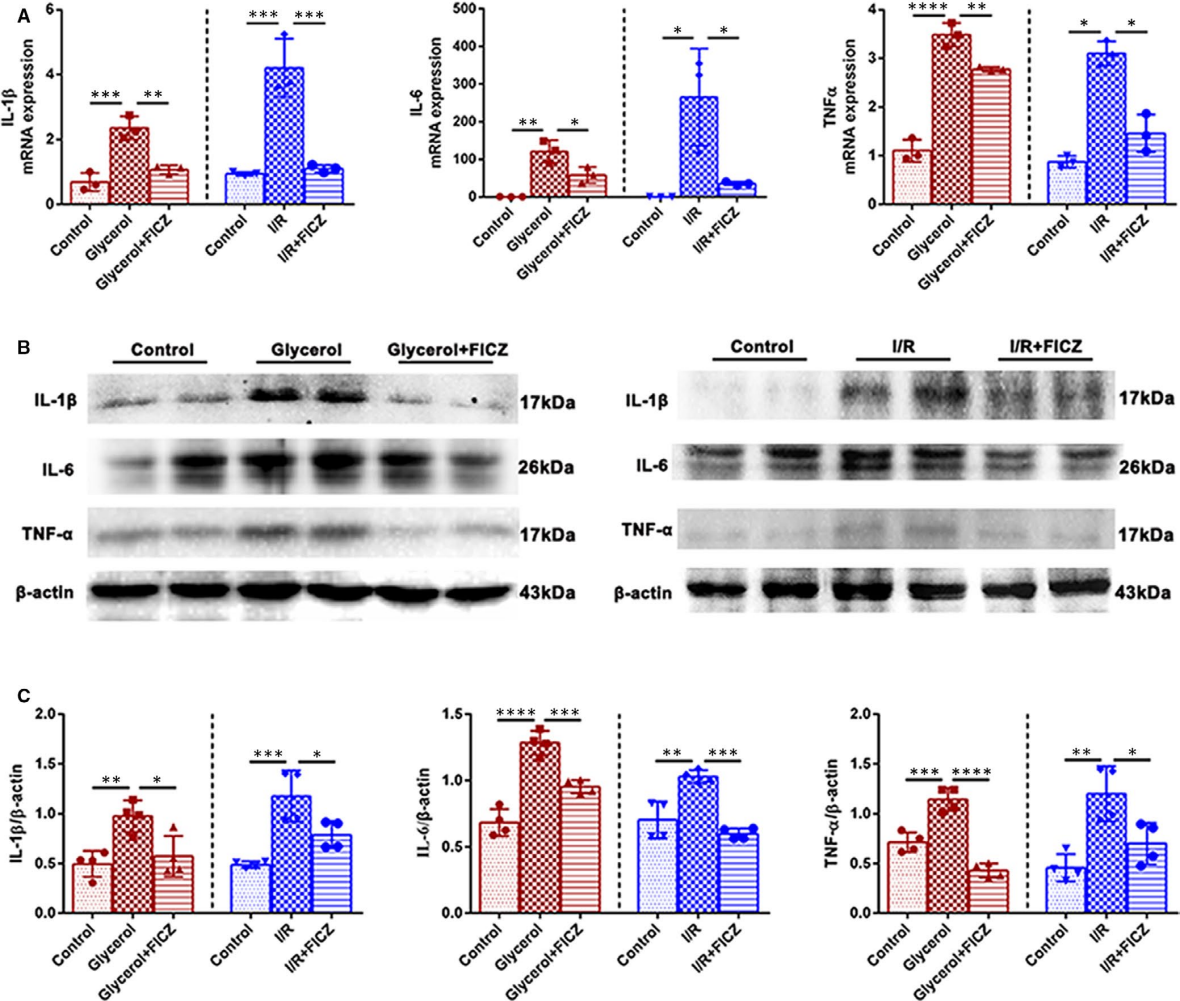
6‐formylindolo[3,2‐b]carbazole (FICZ) alleviated renal inflammation in AKI. A, Quantitative real‐time PCR analysis of IL‐1β, IL‐6 and TNF‐α in renal tissues. B, Immunoblot analysis of IL‐1β, IL‐6, TNF‐α and C, quantifications by densitometry normalized with β‐actin in kidneys. ****P < 0.0001, ***P < 0.001, **P < 0.01, *P < 0.05

To evaluate whether FICZ was able to revolve inflammation evoked by AKI, q‐PCR was used to detect the transcription levels of pro‐inflammatory factors in kidneys. The mRNA levels of IL‐1β, IL‐6 and TNF‐α in renal tissues remarkably increased by glycerol injection or I/R injury, while FICZ administration significantly reduced these gene transcriptions (Figure 4A). Consistently, AKI modelling‐induced the elevation of IL‐1β, IL‐6 and TNF‐α were effectively attenuated by FICZ treatment (Figure 4B‐C). These results suggested that FICZ could alleviate renal inflammation in AKI modelling.

### 3.3 FICZ reduced cell apoptosis in the kidneys of AKI mice

**Figure 5:**
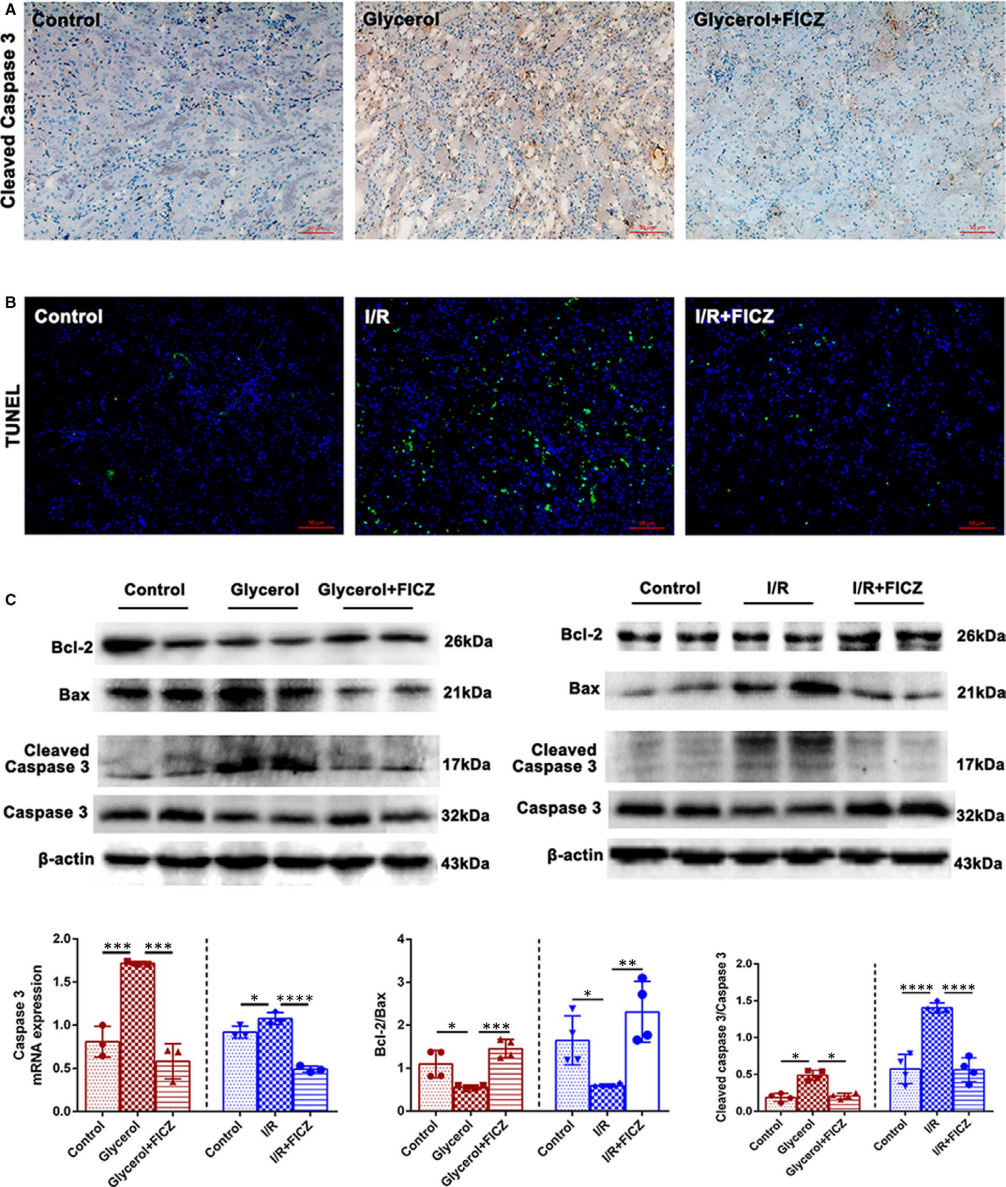
6‐formylindolo[3,2‐b]carbazole (FICZ) decreased cell apoptosis in AKI. A, Immunochemistry staining (×200) of cleaved caspase 3 in kidney tissue sections in rhabdomyolysis‐induced AKI. B, TUNEL staining (×200) of kidney tissue sections in I/R‐induced AKI. C, Immunoblot analysis and quantifications by densitometry of Bcl‐2, BAX, cleaved Caspase 3 in rhabdomyolysis and I/R‐induced AKI. Quantitative real‐time PCR analysis of Caspase 3 in kidneys in rhabdomyolysis and I/R‐induced AKI. ****P < 0.0001, ***P < 0.001, **P < 0.01, *P < 0.05

In addition to inflammation, cell apoptosis is another important feature in the pathogenesis of AKI. We applied TUNEL staining to investigate the effect of FICZ on kidney apoptosis in I/R‐induced mice. As showed in Figure 5B, FICZ significantly reducted the number of TUNEL‐positive cells in the kidneys of I/R mice. Besides, the immunohistochemical staining results exhibited that the expression of cleaved caspase‐3, a vital executioner modifying apoptosis‐related proteins, were significantly reduced by FICZ administration in rhabdomyolysis model (Figure 5A). Meanwhile, FICZ could reduce the mRNA levels of caspase‐3 in both rhabdomyolysis and I/R‐induced AKI models. Additionally, western blot results exhibited that FICZ could down‐regulate the expression of cleaved caspase‐3 and Bax, and up‐regulate the level of Bcl‐2 in the kidneys of AKI mice (Figure 5C). In summary, these findings indicated that FICZ showed anti‐apoptotic effects in the kidneys of AKI mice.

### 3.4 FICZ suppressed NF‐κB and JNK signalling in the kidneys of AKI mice

**Figure 6:**
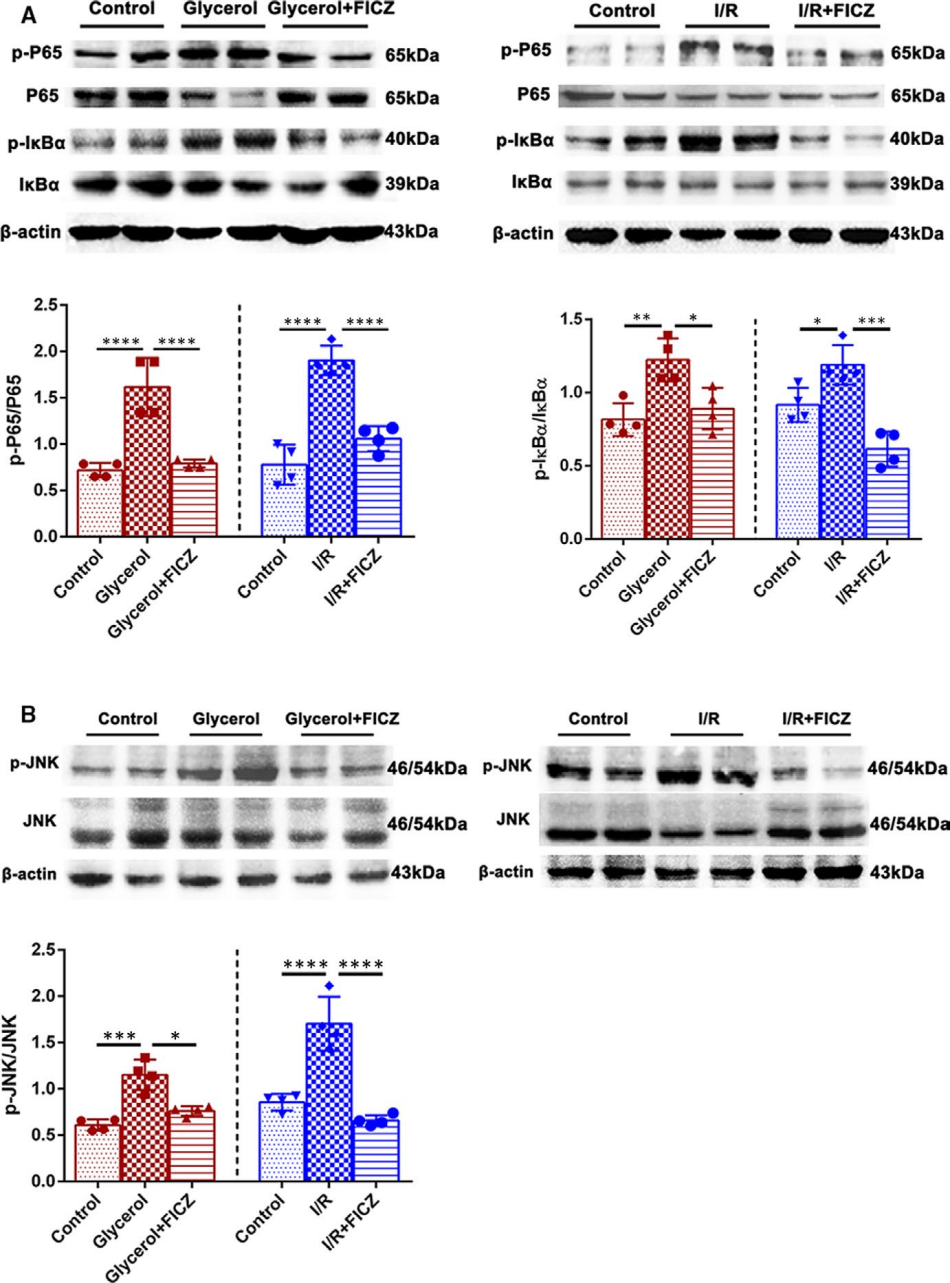
6‐formylindolo[3,2‐b]carbazole (FICZ) suppressed NF‐κB and JNK signaling in the kidneys of AKI mice. A, Immunoblot analysis of p‐IκBα/IκBα, p‐P65/P65 and quantifications by densitometry in rhabdomyolysis and I/R‐induced AKI. B, Immunoblot analysis of p‐JNK/JNK and quantifications by densitometry in rhabdomyolysis and I/R‐induced AKI

The activation of signalling pathways was further examined by western blotting analysis to figure out anti‐inflammatory and anti‐apoptotic mechanism of FICZ in the kidneys of AKI mice. As shown in Figure 6, the phosphorylation levels of IκBα and P65 in the kidneys of AKI mice were obviously higher than those of FICZ‐treated groups. Furthermore, previous studies elucidated that JNK signalling could modulate apoptosis and interact with NF‐κB signalling.^29^ We found that the phosphorylation of JNK were remarkably higher comparing with respective control groups, whereas FICZ treatment effectively reversed them. Taken above, these results implied that the NF‐κB and JNK pathways contributed to the anti‐inflammatory and anti‐apoptotic activity of FICZ in the kidneys of AKI mice.

### 3.5 FICZ protected HK‐2 cells from myoglobin stimulation and H/R injury by inhibiting inflammation and apoptosis

**Figure 7:**
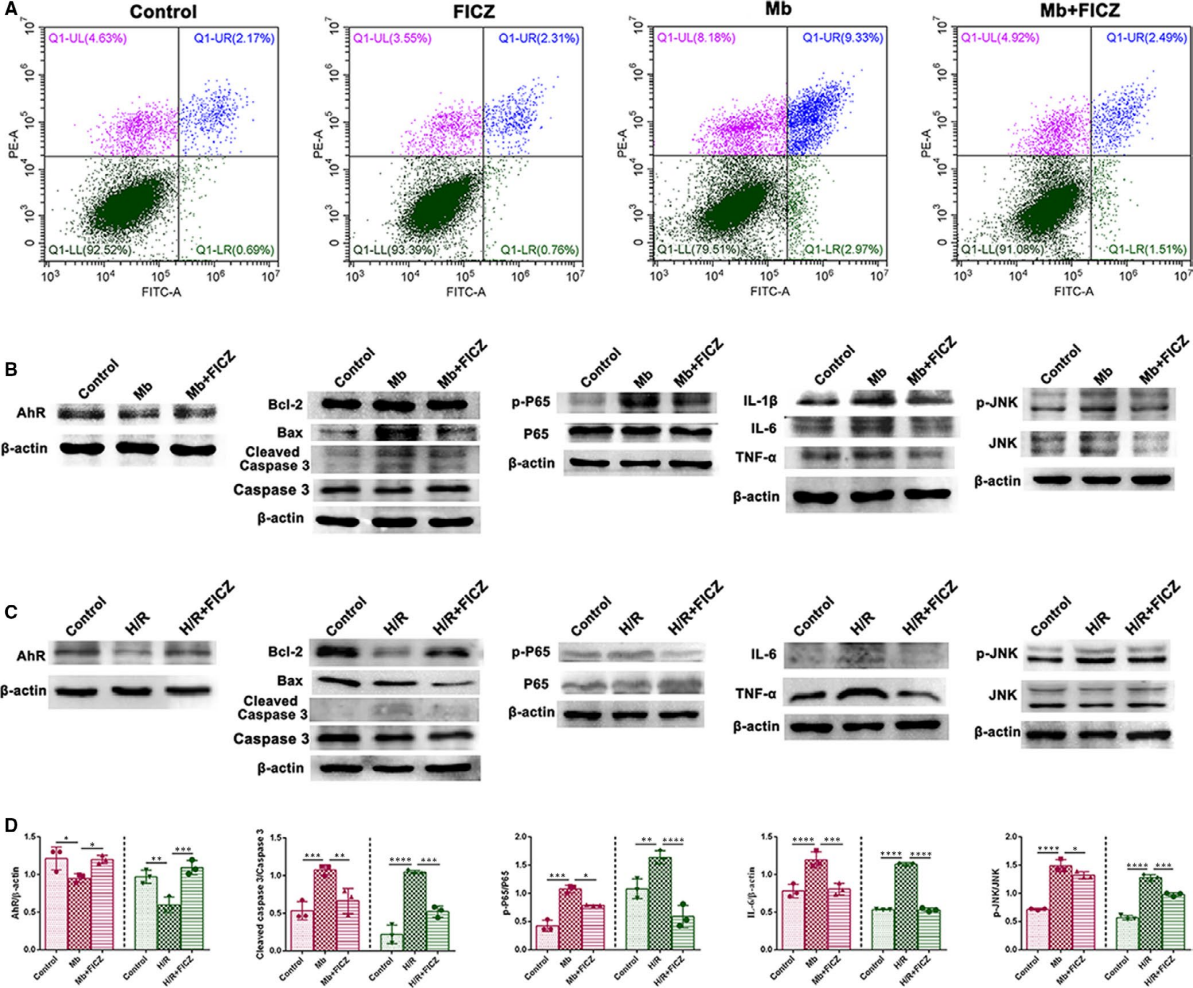
6‐formylindolo[3,2‐b]carbazole (FICZ) alleviated inflammation and apoptosis in myoglobin and H/R‐stimulated HK‐2 cells. A, Flow cytometry in each cell groups. B, Immunoblot analysis in myoglobin model, C, Immunoblot analysis in H/R model, D, Immunoblot analysis of in vitro studies

We testified whether the activation of AhR by FICZ could repress inflammation and apoptosis in myoglobin‐stimulated human proximal tubule epithelial (HK‐2) cells. Firstly, we confirmed that the in vitro expressions of AhR in myoglobin‐stimulated or H/R injured cells were significantly down‐regulated, whereas they were reversed by FICZ (Figure 7B‐D). Cell apoptosis rate of each group in the myoglobin model was detected by flow cytometry (Figure 7A). Comparing with the control group, there was no increase in apoptosis and necrosis of HK‐2 cells in the FICZ group. In contrast, after myoglobin stimulation, HK‐2 cells exhibited a large number of apoptotic cells accounting for 12.3%. While Mb + FICZ group showed significantly less apoptotic cells. In addition, FICZ down‐regulated the expressions of cleaved caspase‐3 and Bax in myoglobin‐stimulated or H/R injured HK‐2 cells (Figure 7B‐D). Furthermore, myoglobin or H/R‐induced the elevation of pro‐inflammatory factors were significantly reversed by FICZ (Figure 7B‐D). Consistently, the phosphorylation levels of P65 and JNK were remarkably higher after myoglobin stimulation or H/R injury, whereas FICZ treatment effectively attenuated them (Figure 7B‐D). Hence, those results indicated that AhR agonist FICZ protected HK‐2 cells against myoglobin and H/R via inhibiting inflammation and apoptosis.

## 4 DISCUSSION

In the current study, we found that AhR agonism by FICZ could attenuate kidney damage in rhabdomyolysis and ischaemia/reperfusion‐induced AKI. AhR was mainly present in proximal tubular epithelial cells, while AhR agonist FICZ could protect proximal tubular epithelial cells against AKI by regulating inflammation and cell apoptosis through NF‐κB and JNK signalling pathways.

Previous studies found that uraemic toxins, like indoxyl sulphate, were agonists of AhR.^30^ Patients with CKD were exposed to those uraemic toxins, thus AhR was activated both in patients and mice with CKD.^25^, ^31^ Recent studies found that endogenous metabolites from gut microbiota^32^ or uraemic metabolism could activate AhR^33^, ^34^ in the kidneys of CKD mice and rats, which exhibited different trends from our findings in AKI mice. However, distinct ligands for AhR could affect different downstream signalling pathways.^35^ In addition, previous research on AKI showed that leflunomide could activate AhR in I/R‐induced AKI and improve renal pathologic damage through regulating immunity and inhibiting inflammation.^36^ MiR‐125b activated AhR via inhibiting AhR repressor and protected kidney from cisplatin‐induced injury.^37^ Baicalin ameliorated aristolochic acid I‐induced kidney injury through AhR‐dependent CYP1A1/2 induction in the liver.^38^ Our results were consistent with these previous findings on AKI. Furthermore, AhR agonist FICZ exerted protective effects in various disease models like metabolic syndrome,^39^ autoimmune uveitis,^22^ colitis,^23^ psoriasis,^24^ etc.

According to recent results of Huang et al,^22^ experimental autoimmune uveitis modelling activated NF‐κB and STAT1/3 pathways, and up‐regulated the expressions of downstream pro‐inflammatory factors IL‐1β, IL‐6 and TNF‐α. Meanwhile, cell apoptosis increased. With AhR agonist treatment, decreased expressions of pro‐inflammatory cytokines and inhibition of NF‐κB pathway were found. AhR activation also led to the inhibition of cell apoptosis. These results were consistent with our experiment. They also found that AhR activation shifted macrophage polarization from M1 towards M2. It suggested that AhR might participate in immune‐regulation, which was another possible explanation for the regulation of downstream pro‐inflammatory cytokines.

There were several reports of a negative correlation between AhR and the RelA (P65) subunit of NF‐κB expression in various cells. Co‐immunoprecipitation was performed with total cell extracts of Hepa1c1c7 cells. Since both AhR and NF‐κB may undergo signal‐dependent nuclear translocation, and ARNT was not found to be associated with P65, thus it was assumed that the association of AhR and P65 was primarily in the cytoplasmic compartment.^40^ The transcriptional activities of the AhR and NF‐κB are modulated by transcriptional coactivators. As previously reported, there was a partial overlap between their coactivators. Consequently, it's conceivable that there may be competition for coactivator binding, such that when one pathway is activated, the other pathway will be repressed through competition for coactivator availability.^41^ The negative correlation was verified again by epigenetics research.^42^ In the meantime, NF‐κB affects with JNK, possibly through reactive oxygen species (ROS).^29^ Mitochondrial ROS could promote NF‐κB activation, and JNK contributes to ROS generation, which in turn stimulates JNK activation. Although the detailed mechanism needs further investigation, it could be assumed that positive correlation between NF‐κB and JNK pathways exists.

Previous research^43^, ^44^ found that in rhabdomyolysis and I/R‐induced AKI, inflammation and cell apoptosis was remarkably increased in the modelling groups, meanwhile NF‐κB and JNK pathways were activated. These findings were consistent with our results. Main mechanisms of AKI included hypoxia, oxidative stress response and inflammation. They could promote cell death (apoptosis, autophagy, necrosis) or tissue remodelling. Cell injury or death released substances with damage‐associated molecular patterns (DAMPs) and pathogen‐associated molecular patterns (PAMPs), which further aggravated inflammation.^45^ Interactions of DAMPs and PAMPs with sensors of the innate immune system also amplified inflammatory response.^46^ In the meantime, cell death including apoptosis also was involved in the pathogenesis of AKI. Caspases family expressions are up‐regulated, and multiple mitochondria‐related anti‐apoptotic and proapoptotic regulators change during AKI.^26^, ^47^, ^48^ Taken together, inflammation and cell apoptosis promote each other and both play vital roles in AKI. We found that AhR activation by FICZ alleviated inflammation and apoptosis via inhibiting NF‐κB and JNK pathways.

However, there are several problems remaining to be investigated. Our study mainly explored the renoprotective effects of AhR activation by FICZ and its possible mechanism in rhabdomyolysis and I/R‐induced AKI. Additionally, we used preventive administration of FICZ to mice before AKI modelling. Although the administration mode was used widely in previous studies,^49^, ^50^, ^51^ discrepancy did exist between the animal experiments and clinical practice. In the future, how expression of AhR changes upon AKI development and more specific intracellular localizations of AhR needs further investigation. While the decrease of NF‐κB and JNK phosphorylation under FICZ action is clear, further experiments of relevant blocking agents are needed. Moreover, to better explain mechanism, AhR knockout and overexpression mice are needed for in vivo study, while overexpression and silence of AhR is needed for in vitro study. In addition, how different ligands affect different AhR signalling pathways needs far more exploration.

## 5 CONCLUSIONS

In summary, AhR agonism by FICZ alleviated rhabdomyolysis and I/R‐induced AKI. FICZ inhibited inflammation and apoptosis via suppressing NF‐κB and JNK signalling pathways in proximal tubular cells.

## CONFLICT OF INTEREST

The authors declare no conflict of interest.

## AUTHOR CONTRIBUTION

**Sibei Tao:** Formal analysis (lead); Investigation (lead); Methodology (lead); Software (lead); Writing‐original draft (lead). **Fan Guo:** Investigation (equal); Methodology (equal). **Qian Ren:** Investigation (equal); Methodology (equal). **Jing Liu:** Methodology (equal). **Tiantian Wei:** Methodology (equal). **Lingzhi Li:** Investigation (equal). **Liang Ma:** Conceptualization (lead); Funding acquisition (equal); Investigation (equal); Supervision (lead); Validation (lead); Writing‐review & editing (lead). **Ping Fu:** Conceptualization (lead); Funding acquisition (lead); Supervision (lead); Writing‐review & editing (equal).

## Supporting information


**Tables S1‐S3**

## Data Availability

The data that support the findings of this study are available from the corresponding author upon reasonable request.
