# Supplementary material for: Activation of aryl hydrocarbon receptor by 6‐formylindolo[3,2‐b]carbazole alleviated acute kidney injury by repressing inflammation and apoptosis
Source: J Cell Mol Med. 2020 Dec 6;25(2):1035–47. doi: 10.1111/jcmm.16168 (PMC7812300; doi:10.1111/jcmm.16168)
Supplement: Supplementary file 1 — Tables S1‐S3 [file JCMM-25-1035-s001.docx]

Supplementary Table 1. Antibodies used in the study.

| Antibody name&species | Company | Cargo number |
| --- | --- | --- |
| Rabbit Anti-Aryl hydrocarbon receptor | Affinity Biosciences | AF6278 |
| Rabbit Anti-phospho-IκBα | Cell Signaling Technology | 2859S |
| Rabbit Anti-IκBα | Cell Signaling Technology | 4812S |
| RabbitAnti- phospho-NF-kB p65 | Cell Signaling Technology | 3033S |
| RabbitAnti- NF-kB p65 | Cell Signaling Technology | 8242S |
| Rabbit Anti-TNF-α | Affinity Biosciences | AF7014 |
| Mouse Anti-IL-6 | Huabio Technology | EM170414 |
| Rabbit Anti-IL-1β | Affinity Biosciences | AF4006 |
| Rabbit Anti- phospho-JNK | Huabio Technology | ET1609-42 |
| Rabbit Anti-JNK | Abcam | ab208035 |
| Rabbit Anti-BAX | Abcam | ab32503 |
| Rabbit Anti-Bcl-2 | Cell Signaling Technology | 3498S |
| Rabbit Anti-Cleaved caspase-3 | Proteintech Group | 66470-2-Ig |
| Rabbit Anti-NGAL | Abcam | ab63929 |
| Rabbit anti-β-actin | Zenbioscience | EE0806 |

Supplementary Table 2. Primer sequences used in the study.

| Gene | Sequences |
| --- | --- |
| β-actin | Forward5’-TCCATCATGAAGTGTGACGT-3’  Reverse5’-GAGCAATGATCTTGATCTTCAT-3’ |
| NGAL | Forward5’-GCAGGTGGTACGTTGTGGG-3’  Reverse5’-CTCTTGTAGCTCATAGATGGTGC-3’ |
| KIM1 | Forward5’-ACATATCGTGGAATCACAACGAC-3’  Reverse5’-ACTGCTCTTCTGATAGGTGACA-3’ |
| AhR | Forward5’-GCCGGTGCAGAAAACAGTAAA-3’  Reverse5’-GGTAACTGACGCTGAGCCTA-3’ |
| IL-1β | Forward5’-CCTCGTGCTGTCGGACCCATA-3’  Reverse5’-CAGGCTTGTGCTCTGCTTGTGA-3’ |
| IL-6 | Forward5’-TAGTCCTTCCTACCCCAATTTCC-3’  Reverse5’-TTGGTCCTTAGCCACTCCTTC-3’ |
| TNF-α | Forward 5’ -GGTGCCTATGTCTCAGCCTCTT-3’  Reverse 5’- GCCATAGAACTGATGAGAGGGAG-3’ |
| Caspase 3 | Forward 5’-TGCTGGTGGGATCAAAGC-3’  Reverse 5’-TGAATCCACTGAGGTTTTGTTG-3’ |
| Bax | Forward 5’-GATCAGCTCGGGCACTTTAG-3’  Reverse 5’-TTGCTGATGGCAACTTCAAC-3’ |
| Bcl-2 | Forward 5’-TGTGAGGACCCAATCTGGAAA-3’  Reverse 5’-TTGCAATGAATCGGGAGTTG-3’ |

Supplementary Table 3. The serum ALT and AST levels between the control and FICZ groups.

|  | Control | FICZ | P value |
| --- | --- | --- | --- |
| ALT (U/L) | 36.41±7.55 | 34.93±8.92 | 0.793 |
| AST (U/L) | 122.99±76.62 | 108.60±21.63 | 0.764 |

ALT: alanine aminitransferase, AST: aspartate aminotransferase.
